# Prevalence and Diagnostic Comparison of *Helicobacter pylori* and Non-*Helicobacter pylori Helicobacter* Infections in Patients Undergoing Upper Gastrointestinal Endoscopy with Gastric Biopsy in Algarve, Portugal

**DOI:** 10.3390/microorganisms13071684

**Published:** 2025-07-17

**Authors:** Francisco Cortez Nunes, Teresa Letra Mateus, Catarina Aguieiras, Ricardo Louro, Bruno Peixe, Mauro Calhindro, Patrícia Queirós, Pedro Castelo-Branco

**Affiliations:** 1 Faculty of Medicine and Biomedical Sciences, University of Algarve, 8005-139 Faro, Portugal; a78540@ualg.pt (F.C.N.); poqueiros@ulsalg.min-saude.pt (P.Q.); pjbranco@ualg.pt (P.C.-B.); 2 CISAS—Center for Research and Development in Agrifood Systems and Sustainability, Escola Superior Agrária, Instituto Politécnico de Viana do Castelo, 4900-367 Viana do Castelo, Portugal; 3 EpiUnit ITR, Instituto de Saúde Pública, Universidade do Porto, 4050-091 Porto, Portugal; 4 Veterinary and Animal Research Centre (CECAV), Associate Laboratory for Animal and Veterinary Sciences (AL4AnimalS), Universidade de Trás-os-Montes e Alto Douro, 5000-801 Vila Real, Portugal; 5 Serviço de Gastroenterologia, Unidade Local de Saúde do Algarve—Hospital de Faro, 8000-386 Faro, Portugal; catarina.maria@edu.ulisboa.pt (C.A.); bpeixe@chalgarve.min-saude.pt (B.P.); 6 Hospital Particular do Algarve—Unidade de Alvor, 8500-322 Portimão, Portugal; louro_scout@hotmail.com; 7 Serviço de Medicina Interna, Unidade Local de Saúde do Algarve—Hospital de Portimão, 8500-338 Portimão, Portugal; mauro.santos@chalgarve.min-saude.pt; 8 Serviço de Gastroenterologia, Unidade Local de Saúde do Algarve—Hospital de Portimão, 8500-338 Portimão, Portugal; 9 Algarve Biomedical Center Research Institute (ABC Ri), University of Algarve, 8005-139 Faro, Portugal

**Keywords:** gastritis, NHPH, *Helicobacter bizzozeronii*, modified Giemsa, PCR

## Abstract

*H. pylori* infects over half of the global population and is associated with various gastric and extra-gastric diseases. Other species, such as zoonotic non-*Helicobacter pylori* Helicobacters (NHPHs), have shown similar associations with gastritis and MALT lymphoma and *H. pylori*-negative cases with gastric disease have been identified, including gastric MALT lymphoma, chronic gastritis, and gastroduodenal ulcers. Accurate identification of these species is of great relevance but remains challenging using conventional diagnostic methods. This cross-sectional study aimed to determine the prevalence of *H. pylori* and NHPH infections, comparing standard histological protocols with molecular techniques. Between December 2024 and February 2025, 54 adult patients undergoing upper gastrointestinal endoscopy (UGE) with gastric biopsy in three hospitals in Algarve, Portugal were recruited. Endoscopic assessment was performed, and gastric biopsies were collected for histological and molecular analysis. DNA was extracted from antral biopsies and analyzed by conventional PCR to detect *H. pylori* and NHPH. *H. pylori* diagnostic techniques were compared, descriptive plus statistical analysis was performed, and *p*-values < 0.05 were considered to be statistically significant. Fifty-four patients were included in the study, with 51.9% of them presenting symptoms. Endoscopic gastritis was observed in 66.7% of patients, while histological gastritis was present in 88.9%, with statistically significant differences between the two diagnostic techniques (*p* = 0.004). *Helicobacter* spp. were identified in 44.4% (24/54) of the patients. *H. pylori* was detected in 42.6% of the patients by Modified Giemsa stain and in 33.3% by PCR. *H. bizzozeronii* was found in 35.9% of the patients, with 22.2% showing mixed infections. This study reveals a significant prevalence of *Helicobacter* spp. in patients from the Algarve region, with both *H. pylori* and zoonotic *H. bizzozeronii* detected. This is the first report of *H. bizzozeronii* DNA detection in gastric biopsies via PCR from patients undergoing UGE in Portugal, highlighting the need to consider NHPH in clinical diagnosis. It is important to include molecular methods in routine diagnostics and the need for broader studies to assess regional and national trends in *Helicobacter* infections besides *H. pylori.*

## 1. Introduction

The *Helicobacter* genus comprises Gram-negative spiral-shaped bacteria, including more than 70 different species that can colonize the gastrointestinal tract of humans and animals and may be associated with both gastric and extra-gastric diseases [1,2,3,4]. *H. pylori* infects over half the global population, causing gastritis, gastroduodenal ulcers, gastric adenocarcinoma, and mucosa-associated lymphoid tissue (MALT) lymphoma [5,6]. *H. pylori* is the most well-known and extensively studied species within this genus. Other gastric helicobacters, collectively referred to as non-*Helicobacter pylori* helicobacters (NHPHs), are also associated with gastrointestinal diseases in humans [3,7,8,9,10] and have gained increasing attention. The most common gastric NHPH in humans are *H. bizzozeronii, H. felis, H*. *suis*, *H. heilmannii*, and *H. salomonis,* all of which have zoonotic potential [7,11]. NHPH infections in humans are less frequent than *H. pylori* infections. Among the NHPH species, *H. bizzozeronii, H. felis*, and *H. suis* are the most prevalent [7]. Although NHPH infections are often associated with milder forms of gastritis, they have been linked to a higher risk of developing low-grade MALT lymphoma compared to *H. pylori* [8,11,12,13]. The primary source of NHPH infections in humans is most likely direct or indirect contact with infected animals and consumption of contaminated food or water [14,15]. *H. bizzozeronii* is considered one of the most frequently detected NHPH species in humans [7,11]. Originally isolated from dogs, *H. bizzozeronii* can colonize the gastric mucosa and is believed to be transmitted primarily through close contact with pets [2,14]. Studies have demonstrated its ability to adhere to human gastric epithelial cells and elicit an inflammatory response similar to *H. pylori,* contributing to chronic gastritis and MALT lymphoma development [7,8,12]. Unlike *H. pylori*, which predominantly affects humans, *H. bizzozeronii* has a broad host range, increasing the risk of zoonotic transmission [2,3]. Clinical differentiation from *H. pylori* is difficult due to overlapping symptoms and histological features, emphasizing the need for targeted molecular diagnostics [11,13]. Recent reports suggest that *H. bizzozeronii* may be underdiagnosed and underreported [9,14].

Several Portuguese studies provide valuable insight into the national epidemiology of *Helicobacter* spp. Bastos et al., 2013 report a very high prevalence of *H. pylori* infection in Portuguese adults, reaching 84.2%, with strong associations to low socioeconomic status, advanced age, and low educational level [16]. Morais et al., 2016 observed a declining trend in *H. pylori* prevalence, particularly among younger age groups, attributing this to improved hygiene, antibiotic use, and healthcare access [17]. In more recent work, Matos et al., 2022 conducted molecular investigations revealing that NHPHs were present in gastric tissue samples from Portuguese patients with a prevalence of 2.5%, as well as emphasized the role of glycan-mediated adhesion mechanisms in colonization of the human gastric mucosa by NHPH, which may contribute to chronic gastritis or even lymphoid tissue activation in *H. pylori*-negative patients [11,12].

Epidemiological studies have shown that NHPH infections are more common than previously thought, with some reports citing prevalence rates as high as 29.1% in symptomatic patients in Europe, as described by Taillieu et al., 2023 [7].

The accurate diagnosis of *Helicobacter* infections requires a combination of clinical suspicion and diagnostic tools, as no single method ensures complete sensitivity and specificity [18]. The clinical diagnosis of *H. pylori* can be conducted using different diagnostic techniques, either noninvasive or invasive [18]. Regarding the most commonly used noninvasive techniques in a clinical setting, the urea breath test (UBT), serological assays, and the stool antigen test can be used, while invasive techniques most commonly used include endoscopy for biopsy-based diagnosis such as histology, culture, and molecular methods and the rapid urease test (RUT) [18]. In patients who undergo upper gastrointestinal endoscopy with gastric biopsy, the diagnosis is routinely based on histological examination often using hematoxylin and eosin or modified Giemsa stain, and immunohistochemical techniques, which are the most widely used invasive methods and serve as the current diagnostic gold standard [19]. However, traditional histological methods may fall short in detecting and distinguishing between *H. pylori* and NHPH due to morphological similarities and low bacterial load. These result in differences regarding the sensitivity of these diagnostic techniques, and they may not allow the identification of the species [7,11], which can result in false-negative and or false-positive results, mainly when using modified Giemsa stain [20]. Molecular methods such as PCR have become increasingly valuable in detecting *Helicobacter* spp. DNA and differentiating between species offering enhanced sensitivity and specificity [7,11]. Recent European studies recommend integrating PCR into diagnostic algorithms, particularly when standard stains are inconclusive or when patients are *H. pylori*-negative despite clinical symptoms [7,11].

From a public health standpoint, understanding the burden of zoonotic *Helicobacter* infections is crucial, particularly in regions where contact with domestic animals or agricultural practices increases exposure risk [9,14]. There is also growing recognition that NHPH infections may contribute to gastric malignancies, especially in patients who test negative for *H. pylori* using conventional diagnostic methods but still present with gastric pathology [8,13]. As such, integrating molecular diagnosis into standard clinical workflows could enhance detection, enable better epidemiological surveillance, and allow more tailored therapeutic approaches [7,21].

Therefore, this study aimed to identify, diagnose, and characterize *H. pylori* and/or NHPH infections to determine the prevalence of *H. pylori* and NHPH infections and to compare the standard protocol for diagnose of *H. pylori* in gastric biopsies using histology and modified Giemsa stain with molecular diagnosis, specifically PCR using species-specific primers for the detection of *Helicobacter* species in patients undergoing upper gastrointestinal endoscopy with gastric biopsy in Algarve, Portugal.

## 2. Materials and Methods

### 2.1. Study Design

A cross-sectional study was carried out at the endoscopy units of three hospitals in Algarve, Portugal (Unidade Local de Saúde do Algarve—Hospital de Faro, Hospital de Portimão, and the Hospital Particular do Algarve—Unidade de Alvor) between December 2024 and February 2025. Patients aged 18 years and older who presented with an indication for upper gastrointestinal endoscopy with gastric biopsy were considered for recruitment. To be included in the study, all patients had to sign a consent form and had to be off proton pump inhibitors and antimicrobials for at least 14 days and 30 days before the procedure, respectively. Informed written consent was obtained from all the participants.

### 2.2. Upper Gastrointestinal Endoscopy and Histological and Molecular Analysis

An upper gastrointestinal endoscopy was performed by a gastroenterologist assisted by a nurse using endoscopes (Olympus^®^ GIF-Q165/185 (Olympus^®^, Tokyo, Japan), Fujifilm^®^ EG-760R (Fujifilm^®^ (Tokyo, Japan)). During the procedure, pictures were taken of the significant findings. Biopsies were taken from the *antrum* and *corpus* for histological analysis and one extra biopsy of the *antrum* was taken for DNA extraction and molecular analysis.

The collected biopsies followed the standard intrahospital diagnostic procedure for histological analysis and *H. pylori* diagnosis with standard modified Giemsa stain. The extra biopsies taken for DNA extraction and molecular analysis were labeled and stored at −20 °C until sent to the Institute for Research and Innovation in Health (i3S)—Porto University for processing and molecular diagnostics.

At the Cell Culture and Genotyping Platform of i3S, DNA was extracted from the frozen biopsies using the conventional salting out protocol. All samples were tested for the presence of *H. pylori*, *H. bizzozeronii*, *H. felis*, *H. suis*, *H. heilmannii*, and *H. salomonis* DNA through conventional PCR using species-specific primers and pre-established protocols as previously described in other studies used in human and animal samples (Table S1).

The amplicons of each PCR-positive sample underwent bidirectional sequencing using the Sanger method at the Genomics Core facility of the Institute of Molecular Pathology and Immunology of the University of Porto, Portugal. Sequence editing and multiple alignments were performed with the MegaX Molecular Evolutionary Genetic Analysis version 10.1.8 [22]. The sequences obtained were subject to BLAST analysis using the non-redundant nucleotide database (http://blast.ncbi.nlm.nih.gov/Blast.cgi (accessed on 24 April 2025)) [23,24].

### 2.3. Data Collection

Each patient was questioned regarding the indication to be admitted for upper gastrointestinal endoscopy and major symptoms at the time of the procedure. For each patient, age, sex, the upper gastrointestinal endoscopy report, and the histology report were consulted, and data were collected regarding endoscopic gastritis classification and diagnosis and histological gastritis classification according to the Updated Sydney System.

### 2.4. Statistical Analysis

The data were analyzed using IBM SPSS^®^ Statistics v30 (IBM Corp., Armonk, NY, USA). Frequencies and descriptive statistics were calculated. For bivariate analysis, Chi-square was applied to verify the statistical significance between groups of data, and Cramer’s V (cv) was used to measure the strength of association between two categorical variables. The McNemar test was applied to dichotomous categorical data. *p*-values < 0.05 were considered to be statistically significant.

## 3. Results

### 3.1. Patients’ Data

A total of 54 patients were included in this study, with a mean age of 51.8 years; 27 of them were males with a mean age of 55.5 years, and 27 were females with a mean age of 48.1 years, as shown in Table 1. All patients were asked about current symptoms at the time of the upper gastrointestinal endoscopy. Most of the patients (51.9%) were symptomatic and the most frequent symptom was epigastralgia (18.5%) followed by dyspepsia (16.7%) and heartburn (11.1%); the most frequent indication for upper gastrointestinal endoscopy was gastric cancer opportunistic screening (38.9%), dyspepsia (16.7%), and heartburn (14.8%), as shown in Table 1.

### 3.2. Upper Gastrointestinal Endoscopic Findings

Of the 54 patients who underwent upper gastrointestinal endoscopy, only 18 (33.3%) presented normal gastric mucosa. Of the 36 patients with endoscopic gastritis, atrophy was the most frequent finding in both male and female patients; however, females presented a higher prevalence of erosion (22.2%) and ulceration (11.1%), as shown in Table 2.

### 3.3. Histology Results

Regarding the histological findings of the analyzed biopsies, there were a total of 48 patients with gastritis. There was a higher prevalence of mild gastritis in both male (74.1%) and female (51.9%) patients, with only one female (3.7%) patient with severe gastritis (shown in Table 3). On histological analysis, there was no dysplasia. Atrophy and intestinal metaplasia ranged from mild to moderate, and activity ranged from mild to severe.

Comparing the endoscopic and histological findings, there were normal endoscopy and histology in 4 patients, endoscopic and histological gastritis in 34 patients, endoscopic gastritis with normal histology in 2 patients, and normal endoscopy with histological gastritis in 14 patients. There was a statistically significant difference (*p* = 0.004) when comparing the endoscopic (36/54) and the histological (48/54) diagnosis of gastritis, with higher diagnosis of gastritis using histological diagnosis.

### 3.4. Presence of Helicobacter spp.

Despite the diagnostic technique used, *Helicobacter* species were identified in 24 of the 54 analyzed samples, and there was a statistically significant mild association between sex and the identification of *Helicobacter* species (as shown in Table 4).

Regarding the presence of *H. pylori* using modified Giemsa stain, there was a total of 23 positive samples, with a statistically significant moderate association between female sex and the identification of the presence of *H. pylori* using modified Giemsa stain (*p* = 0.013, cv = 0.337) (as shown in Table 4).

Considering the presence of *Helicobacter* species through PCR analysis, 20/54 (37.0%) patients were PCR-positive for *Helicobacter* spp. DNA, 18/54 (33.3%) patients were PCR-positive for *H. pylori* DNA, and 14/54 (35.9%) were PCR-positive for *H. bizzozeronii* DNA, as shown in Table 4. Mixed infections with PCR-positive *H. pylori* DNA plus *H. bizzozeronii* DNA were detected in 12/54 (22.2%) patients, 6/54 (11.1%) were PCR-positive just for *H. pylori* DNA, and 2/54 (3.7%) of the patients were PCR-positive only for *H. bizzozeronii* DNA. No amplification of DNA was achieved for the remaining NHPH-tested species, as presented in Table 4.

Regarding the endoscopic findings, histological findings, and the presence of *Helicobacter* spp. (Table 5), there was a statistically significant association between them, as presented in Table 6.

## 4. Discussion

It is known that *Helicobacter* spp. may be associated with gastric disease in humans, and it is well-known that *H. pylori* is associated with gastritis, gastroduodenal ulcers, gastric adenocarcinoma, and MALT lymphoma [6].

At the time of the upper gastrointestinal endoscopy, 51.9% of the patients were symptomatic, with the most frequent symptom being epigastralgia (18.5%), followed by dyspepsia (16.7%) and heartburn (11.1%). Our results showed that 66.7% (36/54) of the patients had endoscopic gastritis; however, 88.9% (48/54) had histological gastritis with a statistically significant difference between these two methods (*p* = 0.004), as shown in Table 2 and Table 3, suggesting that endoscopic and histological results are not equivalent. These results corroborate the use of histology as the gold standard method for the diagnosis of antral gastritis, and biopsies should always be performed, regardless of the endoscopic findings, as stated by Bertges et al., 2018 [25].

This study reports a prevalence of *Helicobacter* spp. infection of 44.4% (24 of 54 patients), a lower prevalence compared with the Portuguese prevalence of 84.2% to 90% reported by Bastos et al., 2013 and Morais et al., 2016 [16,17]; however, it is similar to the worldwide studies’ prevalence of 43.1–44.3% [26,27]. The discrepancy with older Portuguese data could reflect improved hygiene, antibiotic usage, and public health measures or may results from sample size limitation since our study reports regional prevalence with a small study population.

Interestingly, the prevalence had a statistically significant moderate association with sex (*p* = 0.028, cv = 0.298), with females presenting a prevalence of *Helicobacter* infections of 59.3% compared to 29.6% in male patients. Although the mechanism underlying sex differences in infection prevalence are unclear, previous studies suggest that horminal, immunological and behavioral factors may contribute [27,28,29,30]. No direct comparison can be made, since our study refers to prevalence of *H. pylori* and *H. bizzozeronii*; however, there are some studies referring to prevalences of *H. pylori* infection ranging from 29.2 to 42.7% in females and 36.6–46.3% in males [27,28,29,30], although these studies included a higher number of male than female participants. Further studies must be conducted to determine how sex influences the acquisition of *Helicobacter* spp. infections.

Regarding the presence of NHPH, only DNA of the zoonotic *H. bizzozeronii* was detected, with a prevalence of 25.9% (14/54). The prevalence was also higher in females (37.0%, 10/27) than in males (14.8%, 4/27). To the best of the authors’ knowledge, this finding is significant as it represents the first report of *H. bizzozeronii* DNA detection in gastric biopsies from patients undergoing upper gastrointestinal endoscopy in Portugal. The fact that only *H. bizzozeronii* DNA was detected goes along with previous reports that state that this is one of the most common NHPHs to infect humans [7].

When comparing the diagnostic techniques to diagnose *H. pylori* infection, namely histology using modified Giemsa (23/54) vs. PCR (18/54), there was a statistically significant strong association between the two techniques (*p* < 0.001, cv = 0.741). These findings are consistent with the literature, suggesting that while modified Giemsa is a reliable method, it lacks the species-level specificity and sensitivity of molecular methods, particularly in the context of NHPH detection [30,31]. Moalla et al., 2024 report 81.5% sensitivity and 56.3% specificity of the modified Giemsa stain; however, Matos et al., 2022 and Taillieu et al., 2023 explain that despite gastric bacterial load, PCR is more sensitive and more specific since it allows for species differentiation. Despite these reports suggesting differences in histology diagnosis compared to molecular techniques [7,11,21,31], our study suggests that histology can be considered an efficient test compared to PCR in *H. pylori* detection, as reported by Moalla et al., 2024 [21]; however, it cannot identify the species and is dependent on the expertise of the pathologist and awareness for morphological differences between *H. pylori* and NHPHs.

When analyzing the presence of gastritis (48/54) with the presence of *Helicobacter* spp. (24/54), there were 25 patients with histological gastritis who were negative for *Helicobacter*, 23 patients with histological gastritis who were positive for *Helicobacter*, and only one patient who was negative for histological gastritis and PCR-positive for *Helicobacter*. Nonetheless, no statistically significant association was found between these two variables (*p* = 0.146). This may suggest that gastritis can have multifactorial etiologies and may occur independently of detectable *Helicobacter* infections. Yet, there was a statistically significant moderate association between the presence of *Helicobacter* spp. and ulceration on endoscopy (*p* = 0.042, cv= 0.280) and a statistically significant strong association between the presence of *Helicobacter* spp. and the severity of gastritis according to the Updated Sydney System Classification (*p* < 0.001, cv = 0.582), as shown in Table 5, as previous reports suggest [32,33].

## 5. Conclusions

This was the first study, at the national level, to compare the standard diagnostic protocol for *H. pylori* with molecular diagnostics, as well as the first to detect *H. bizzozeronii* DNA in gastric samples of patients undergoing UGE with gastric biopsy. The study concludes that NHPHs, in this case, *H. bizzozeronii*, are often associated with gastritis along with *H. pylori*, underlining the clinical importance of considering NHPHs in routine diagnostic workflows and contributing to the emerging epidemiological picture of zoonotic *Helicobacter* infections in Southern Portugal. These findings support the inclusion of PCR-based methods into standard diagnostic algorithms to enhance sensitivity and allow for species-level identification, which is crucial for appropriate clinical management. Ideally, this study should be replicated with a larger number of participants and in other regions of Portugal to determine whether regional differences exist in the prevalence of *H. pylori* and NHPH infections. Moreover, longitudinal studies could provide insight into the chronic outcomes of NHPH infections and their role in gastric disease.

## Figures and Tables

**Table 1:** Clinical characteristics of patients per sex.

	Total	Male	Female
***N***	54	27 (50%)	27 (50%)
	*n* (%)	*n* (%)	*n* (%)
**Age**			
Mean (± SD)	51.81 (±15.80)	55.52 (±13.75)	48.11 (±17.08)
Median [IQR]	51.00 [25.75]	57.00 [18.00]	48.00 [28.00]
**Indication for UGE**			
Gastric cancer opportunistic screening	21 (38.9%)	12 (44.4%)	9 (33.3%)
Dyspepsia	9 (16.7%)	3 (11.1%)	6 (22.2%)
Heartburn	8 (14.8%)	2 (7.4%)	6 (22.2%)
Pre surgical assessment	7 (13.0%)	3 (11.1%)	4 (14.8%)
Epigastralgia	6 (11.1%)	4 (14.8%)	2 (7.4%)
Nausea	1 (1.9%)	1 (3.7%)	0 (0.0%)
Dysphagia	1 (1.9%)	1 (3.7%)	0 (0.0%)
Diarrhea	1 (1.9%)	1 (3.7%)	0 (0.0%)
**Symptomatic**	28 (51.9%)	11 (40.7%)	17 (63.0%)
**Symptoms**			
Epigastralgia	10 (18.5%)	4 (14.8%)	6 (22.2%)
Dyspepsia	9 (16.7%)	3 (11.1%)	6 (22.2%)
Heartburn	6 (11.1%)	1 (3.7%)	5 (18.5%)
Nausea	1 (1.9%)	1 (3.7%)	0 (0.0%)
Dysphagia	1 (1.9%)	1 (3.7%)	0 (0.0%)
Bloating	1 (1.9%)	1 (3.7%)	0 (0.0%)
**Endoscopic gastritis**	36 (66.7%)	18 (66.7%)	18 (66.7%)
**Histological gastritis**	48 (88.9%)	24 (88.9%)	24 (88.9%)
***Helicobacter*** **-positive**	24 (44.4%)	8 (29.6%)	16 (59.3%)
***H. pylori*** **-positive on Modified Giemsa stain**	23 (42.6%)	7 (25.9%)	16 (59.3%)
***H. pylori*** **PCR-positive**	18 (33.3%)	7 (25.9%)	11 (40.7%)
**NHPH** **PCR-positive**	14 (25.9%)	4 (14.8%)	10 (37.0%)

**Table 2:** Endoscopic findings per sex.

	Total	Male	Female
***N***	54	27	27
	*n* (%)	*n* (%)	*n* (%)
**Endoscopic findings**			
**Gastritis**	36 (66.7%)	18 (66.7%)	18 (66.7%)
**Atrophy**	10 (18.5%)	5 (18.5%)	5 (18.5%)
**Erosion**	9 (16.7%)	3 (11.1%)	6 (22.2%)
**Ulceration**	3 (5.6%)	0 (0.0%)	3 (11.1%)
**Marble appearance**	3 (5.6%)	2 (7.4%)	1 (3.7%)

**Table 3:** Histological findings per sex.

	Total	Male	Female
***N***	54	27	27
	*n* (%)	*n* (%)	*n* (%)
**Histological gastritis**	48 (88.9%)	24 (88.9%)	24 (88.9%)
**Updated Sydney System Classification and grading of gastritis**			
Absent	6 (11.1%)	3 (11.1%)	3 (11.1%)
Mild	34 (63.0%)	20 (74.1%)	14 (51.9%)
Moderate	13 (24.1%)	4 (14.8%)	9 (33.3%)
Severe	1 (1.9%)	0 (0.0%)	1 (3.7%)
*Helicobacter* spp. Modified Giemsa- or PCR-Positive	24 (44.4%)	8 (29.6%)	16 (59.3%)

**Table 4:** *Helicobacter* spp.-positive samples per sex and diagnostic technique.

	Total	Male	Female	
***N***	54	27	27	
	*n* (%)	*n* (%)	*n* (%)	
***Helicobacter*** **spp.** **Modified Giemsa- and PCR-Positive**	24 (44.4%)	8 (29.6%)	16 (59.3%)	*p* = 0.028 cv = 0.298
***H. pylori*** **Modified Giemsa-Positive**	23 (42.6%)	7 (25.9%)	16 (59.3%)	*p* = 0.013 cv = 0.337
***H. pylori*** **PCR-positive**	18 (33.3%)	7 (25.9%)	11 (40.7%)	
**NHPH** **PCR-positive**	14 (25.9%)	4 (14.8%)	10 (37.0%)	
***H. bizzozeronii*** **PCR-positive**	14 (25.9%)	4 (14.8%)	10 (37.0%)	
***H. felis*** **PCR-positive**	0 (0.0%)	0 (0.0%)	0 (0.0%)	
***H. suis*** **PCR-positive**	0 (0.0%)	0 (0.0%)	0 (0.0%)	
***H. heilmannii*** **PCR-positive**	0 (0.0%)	0 (0.0%)	0 (0.0%)	
***H. salomonis*** **PCR-positive**	0 (0.0%)	0 (0.0%)	0 (0.0%)	

**Table 5:** Percentage of gastritis and presence of *Helicobacter* spp. per diagnostic technique.

		Gastritis		*Helicobacter* spp.		
		Endoscopic Diagnosis	Histological Diagnosis	*H. pylori*-Positive Modified Giemsa Stain	*H. pylori*-Positive PCR	*H. bizzozeronii*-Positive PCR
	***N***	***n*** **(%)**	***n*** **(%)**	***n*** **(%)**	***n*** **(%)**	***n*** **(%)**
**Total**	54	48 (88.9%)	36 (66.7%)	23 (42.6%)	18 (33.3%)	14 (25.9%)

**Table 6:** Association between presence of *Helicobacter* spp. and endoscopic and histological findings.

	Ulceration on Endoscopy	Updated Sydney System Classification and Grading of Gastritis
***Helicobacter* ** **spp.**	*p* = 0.042 cv = 0.280	*p* < 0.001 cv = 0.582
***H. bizzozeronii***		*p* < 0.001 cv = 0.627

## Data Availability

The original contributions presented in this study are included in the article/Supplementary Materials. Further inquiries can be directed to the corresponding author.

## Supplementary Materials

The following supporting information can be downloaded at: https://www.mdpi.com/article/10.3390/microorganisms13071684/s1. Table S1: Primer sequences and thermocycling conditions for *H. pylori* and NHPH.

## References

[1] Parte A.C., Sardà Carbasse J., Meier-Kolthoff J.P., Reimer L.C., Göker M. (2020). List of Prokaryotic names with Standing in Nomenclature (LPSN) moves to the DSMZ. Int. J. Syst. Evol. Microbiol..

[2] Haesebrouck F., Pasmans F., Flahou B., Chiers K., Baele M., Meyns T., Decostere A., Ducatelle R. (2009). Gastric helicobacters in domestic animals and nonhuman primates and their significance for human health. Clin. Microbiol. Rev..

[3] Flahou B., Haesebrouck F., Smet A., Backert S., Yamaoka Y. (2016). Non-Helicobacter pylori Helicobacter Infections in Humans and Animals. Helicobacter pylori Research: From Bench to Bedside.

[4] Baj J., Forma A., Flieger W., Morawska I., Michalski A., Buszewicz G., Sitarz E., Portincasa P., Garruti G., Flieger M. (2021). *Helicobacter pylori* Infection and Extragastric Diseases-A Focus on the Central Nervous System. Cells.

[5] Katelaris P., Hunt R., Bazzoli F., Cohen H., Fock K.M., Gemilyan M., Malfertheiner P., Mégraud F., Piscoya A., Quach D. (2023). *Helicobacter pylori* World Gastroenterology Organization Global Guideline. J. Clin. Gastroenterol..

[6] Malfertheiner P., Camargo M.C., El-Omar E., Liou J.M., Peek R., Schulz C., Smith S.I., Suerbaum S. (2023). *Helicobacter pylori* infection. Nat. Rev. Dis. Primers.

[7] Taillieu E., De Witte C., De Schepper H., Van Moerkercke W., Rutten S., Michiels S., Arnst Y., De Bruyckere S., Francque S., van Aert F. (2023). Clinical significance and impact of gastric non-*Helicobacter pylori Helicobacter* species in gastric disease. Aliment. Pharmacol. Ther..

[8] Yasuda T., Lee H.S., Nam S.Y., Katoh H., Ishibashi Y., Yamagata Murayama S., Matsui H., Masuda H., Rimbara E., Sakurazawa N. (2022). Non-*Helicobacter pylori Helicobacter* (NHPH) positive gastric cancer. Sci. Rep..

[9] Matos R., Amorim I., Magalhães A., Haesebrouck F., Gärtner F., Reis C.A. (2021). Adhesion of *Helicobacter* Species to the Human Gastric Mucosa: A Deep Look Into Glycans Role. Front. Mol. Biosci..

[10] Ali B., Chloë W., Mehmet A., Sofie B., Annemieke S., Gökhan T., Tülin G.G., Freddy H., Fatih K. (2018). Presence of gastric *Helicobacter* species in children suffering from gastric disorders in Southern Turkey. Helicobacter.

[11] Matos R., Taillieu E., De Bruyckere S., De Witte C., Rêma A., Santos-Sousa H., Nogueiro J., Reis C.A., Carneiro F., Haesebrouck F. (2022). Presence of Helicobacter Species in Gastric Mucosa of Human Patients and Outcome of *Helicobacter* Eradication Treatment. J. Pers. Med..

[12] Matos R., Sousa H.S., Nogueiro J., Magalhães A., Reis C.A., Carneiro F., Amorim I., Haesebrouck F., Gärtner F. (2022). *Helicobacter* species binding to the human gastric mucosa. Helicobacter.

[13] Takigawa H., Yuge R., Masaki S., Otani R., Kadota H., Naito T., Hayashi R., Urabe Y., Oka S., Tanaka S. (2021). Involvement of non-Helicobacter pylori helicobacter infections in *Helicobacter pylori*-negative gastric MALT lymphoma pathogenesis and efficacy of eradication therapy. Gastric. Cancer.

[14] Mladenova-Hristova I., Grekova O., Patel A. (2017). Zoonotic potential of *Helicobacter* spp. J. Microbiol. Immunol. Infect..

[15] Youssef A.I., Afifi A., Abbadi S., Hamed A., Enany M. (2021). PCR-based detection of *Helicobacter pylori* and non-*Helicobacter pylori* species among humans and animals with potential for zoonotic infections. Pol. J. Vet. Sci..

[16] Bastos J., Peleteiro B., Barros R., Alves L., Severo M., de Fátima Pina M., Pinto H., Carvalho S., Marinho A., Guimarães J.T. (2013). Sociodemographic determinants of prevalence and incidence of *Helicobacter pylori* infection in Portuguese adults. Helicobacter.

[17] Morais S., Ferro A., Bastos A., Castro C., Lunet N., Peleteiro B. (2016). Trends in gastric cancer mortality and in the prevalence of *Helicobacter pylori* infection in Portugal. Eur. J. Cancer Prev..

[18] Sousa C., Ferreira R., Santos S.B., Azevedo N.F., Melo L.D.R. (2023). Advances on diagnosis of *Helicobacter pylori* infections. Crit. Rev. Microbiol..

[19] García-Morales N., Pérez-Aísa Á., Fiorini G., Tepes B., Castro-Fernández M., Lucendo A., Voynovan I., Bujanda L., Garre A., Rodrigo L. (2023). *Helicobacter pylori* Diagnostic Tests Used in Europe: Results of over 34,000 Patients from the European Registry on Helicobacter pylori Management. J. Clin. Med..

[20] Khan H., Rauf F., Muhammad N., Javaid M., Alam S., Nasir S. (2022). Comparison of special stains (Giemsa stain and Modified Toluidine Blue stain) with immunohistochemistry as gold standard for the detection of *H. pylori* in gastric biopsies. Arab. J. Gastroenterol..

[21] Moalla M., Chtourou L., Mnif B., Charfi S., Smaoui H., Boudabous M., Mnif L., Amouri A., Gdoura H., Hammami A. (2024). Assessment of histology’s performance compared with PCR in the diagnosis of *Helicobacter pylori* infection. Future Sci. OA.

[22] Stecher G., Tamura K., Kumar S. (2020). Molecular Evolutionary Genetics Analysis (MEGA) for macOS. Mol. Biol. Evol..

[23] Altschul S.F., Gish W., Miller W., Myers E.W., Lipman D.J. (1990). Basic local alignment search tool. J. Mol. Biol..

[24] Benson D.A., Karsch-Mizrachi I., Lipman D.J., Ostell J., Rapp B.A., Wheeler D.L. (2002). GenBank. Nucleic Acids Res..

[25] Bertges L.C., Dibai F.N., Bezerra G., Oliveira E.S., Aarestrup F.M., Bertges K.R. (2018). Comparison between the endoscopic findings and the histological diagnosis of antral gastritis. Arq. Gastroenterol..

[26] Li Y., Choi H., Leung K., Jiang F., Graham D.Y., Leung W.K. (2023). Global prevalence of *Helicobacter pylori* infection between 1980 and 2022: A systematic review and meta-analysis. Lancet Gastroenterol. Hepatol..

[27] Zamani M., Ebrahimtabar F., Zamani V., Miller W.H., Alizadeh-Navaei R., Shokri-Shirvani J., Derakhshan M.H. (2018). Systematic review with meta-analysis: The worldwide prevalence of Helicobacter pylori infection. Aliment. Pharmacol. Ther..

[28] de Martel C., Parsonnet J. (2006). *Helicobacter pylori* Infection and Gender: A Meta-Analysis of Population-Based Prevalence Surveys. Dig. Dis. Sci..

[29] Ibrahim A., Morais S., Ferro A., Lunet N., Peleteiro B. (2017). Sex-differences in the prevalence of *Helicobacter pylori* infection in pediatric and adult populations: Systematic review and meta-analysis of 244 studies. Dig. Liver Dis..

[30] Almashhadany D.A., Mayas S.M., Mohammed H.I., Hassan A.A., Khan I.U.H. (2023). Population- and Gender-Based Investigation for Prevalence of *Helicobacter pylori* in Dhamar, Yemen. Can. J. Gastroenterol. Hepatol..

[31] Kisa O., Albay A., Refik Mas M., Celasun B., Doganci L. (2002). The evaluation of diagnostic methods for the detection of *Helicobacter pylori* in gastric biopsy specimens. Diagn. Microbiol. Infect. Dis..

[32] Şenocak Taşçı E., Akbaş T. (2020). The Relationship between the Sydney Classification and the First-Line Treatment Efficacy in *Helicobacter*-Associated Gastritis. Med. Princ. Pract..

[33] Anjana M.L., Kavitha Y. (2021). Evaluation of chronic gastritis with *Helicobacter pylori* using updated Sydney system. Int. J. Res. Med. Sci..

