# Supplementary material for: Prevalence and Diagnostic Comparison of Helicobacter pylori and Non-Helicobacter pylori Helicobacter Infections in Patients Undergoing Upper Gastrointestinal Endoscopy with Gastric Biopsy in Algarve, Portugal
Source: Microorganisms. 2025 Jul 17;13(7):1684. doi: 10.3390/microorganisms13071684 (PMC12298905; doi:10.3390/microorganisms13071684)
Supplement: Supplementary file 1 [file microorganisms-13-01684-s001.zip › microorganisms-3750588-supplementary.pdf]

microorganisms

Table S1. Primer sequences and thermocycling conditions for *H. pylori* and NHPH.

***Helicobacter* species**	**Primer**	**Sequence**	**Target gene**	**Amplicon size**	**Thermocycling conditions**			***Helicobacter* strain positive control**
					**Nr. Cycles**	**Temp. (ºC)**	**Time**	
H. suis	BFHsuis_F1	AAA ACA MAg gCg ATC gCC CTg TA	*Urea*	150bp	40	95<br>60<br>72	20 sec<br>30 sec<br>30 sec	HS1
	BFHsuis_R1	TTT CTT CgC CAg gTT CAA AgC g	*urea*					
H. heilmannii	Hh-IceA-FWQ	gTT TCC AAC CAA AAg ACT CA	*iceA*	135 bp	30	94<br>55<br>72	30 sec<br>30 sec<br>30 sec	ASB1.4
	Hh-IceA-RVQ	ATT gCC TAg Agg TTg TgT Tg	*iceA*					
H. salomonis	Hsal_FQ_PAR	CTC TTA TgA gTT ggA CTT ggT gCT CAC CAA T	*ureAB*	91 bp	45	94<br>61<br>72	30 sec<br>30 sec<br>1 min	R1051
	Hsal_RQ_PAR	TTT gCC ATC TTT AAT TCC AAT gTC ggC	*ureAB*					
H. felis	BFHfel_F2	gCT ggT ggC ATC gAT ACg CAT	*ureAB*	154 bp	45	94<br>60<br>72	30 sec<br>30 sec<br>1 min	CS1
	BFHfel_R2	TTT TTA gAT TAg CgC gTC Cgg gA	*ureAB*					
H. bizzozeronii	Hbizz_FQ_PAR	CCA ACA AAT CCC CAC AgC ATT TgC CAg	*ureAB*	91 bp	45	94<br>58<br>72	1 min<br>1 min<br>1 min	R1053
	Hbizz_RQ_PAR	AgT CCC ATC AgC Wgg WCC TgT TCC CCC AC	*ureAB*					
H. pylori	Hpy3F	TTATCGGTAAAGACACC AGAAA	*glmM*	144	45	94<br>54<br>72	30 sec<br>30 sec<br>30 sec	SS1
	Hpy3R	ATCACAGCGCATGTCTTC	*glmM*					
